# Supplementary material for: Mechanisms governing the pioneering and redistribution capabilities of the non-classical pioneer PU.1
Source: Nat Commun. 2020 Jan 21;11:402. doi: 10.1038/s41467-019-13960-2 (PMC6972792; doi:10.1038/s41467-019-13960-2)
Supplement: Supplementary file 7 — Source data [file 41467_2019_13960_MOESM7_ESM.zip › Source_Data/Figure6/Figure6B_MotifScanOutput/FLI1_PU.1/homerResults/motif17.similar.html]

motif17

## Information for motif17

G
T
A
C
G
A
T
C
C
A
T
G
T
G
A
C
A
G
T
C
A
G
T
C
G
T
C
A
G
T
A
C
C
G
T
A
  
Reverse Opposite:  

G
C
A
T
C
A
T
G
A
C
G
T
C
T
A
G
A
C
T
G
A
C
T
G
G
T
A
C
C
T
A
G
C
A
T
G
  

|  |  |
| --- | --- |
| p-value: | 1e-103 |
| log p-value: | -2.378e+02 |
| Information Content per bp: | 1.690 |
| Number of Target Sequences with motif | 2722.0 |
| Percentage of Target Sequences with motif | 27.36% |
| Number of Background Sequences with motif | 6335.8 |
| Percentage of Background Sequences with motif | 17.36% |
| Average Position of motif in Targets | 100.0 +/- 55.0bp |
| Average Position of motif in Background | 98.9 +/- 65.2bp |
| Strand Bias (log2 ratio + to - strand density) | 0.0 |
| Multiplicity (# of sites on avg that occur together) | 1.16 |
| Motif File: | file (matrix) reverse opposite |

### Similar de novo motifs found

|  |  |  |  |  |  |  |  |
| --- | --- | --- | --- | --- | --- | --- | --- |
| Rank | Match Score | Redundant Motif | P-value | log P-value | % of Targets | % of Background | Motif file |
| 1 | 0.961 | G T A C A C T G A G T C A G T C G A T C G T C A G T A C C G T A | 1e-91 | -210.805461 | 25.96% | 16.71% | motif file (matrix) |
| 2 | 0.844 | C T G A C T A G A C T G A T C G G A C T A C T G A C T G A C T G A C T G A C T G C G T A A C T G A C T G | 1e-19 | -45.484198 | 2.46% | 1.14% | motif file (matrix) |
